# Supplementary material for: Coffea arabica Extracts and Metabolites with Potential Inhibitory Activity of the Major Enzymes in Bothrops asper Venom
Source: Pharmaceuticals (Basel). 2025 Aug 1;18(8):1151. doi: 10.3390/ph18081151 (PMC12389448; doi:10.3390/ph18081151)
Supplement: Supplementary file 1 [file pharmaceuticals-18-01151-s001.zip › pharmaceuticals-3731967-supplementary.pdf]

pharmaceuticals

MDPI

Supplementary Material

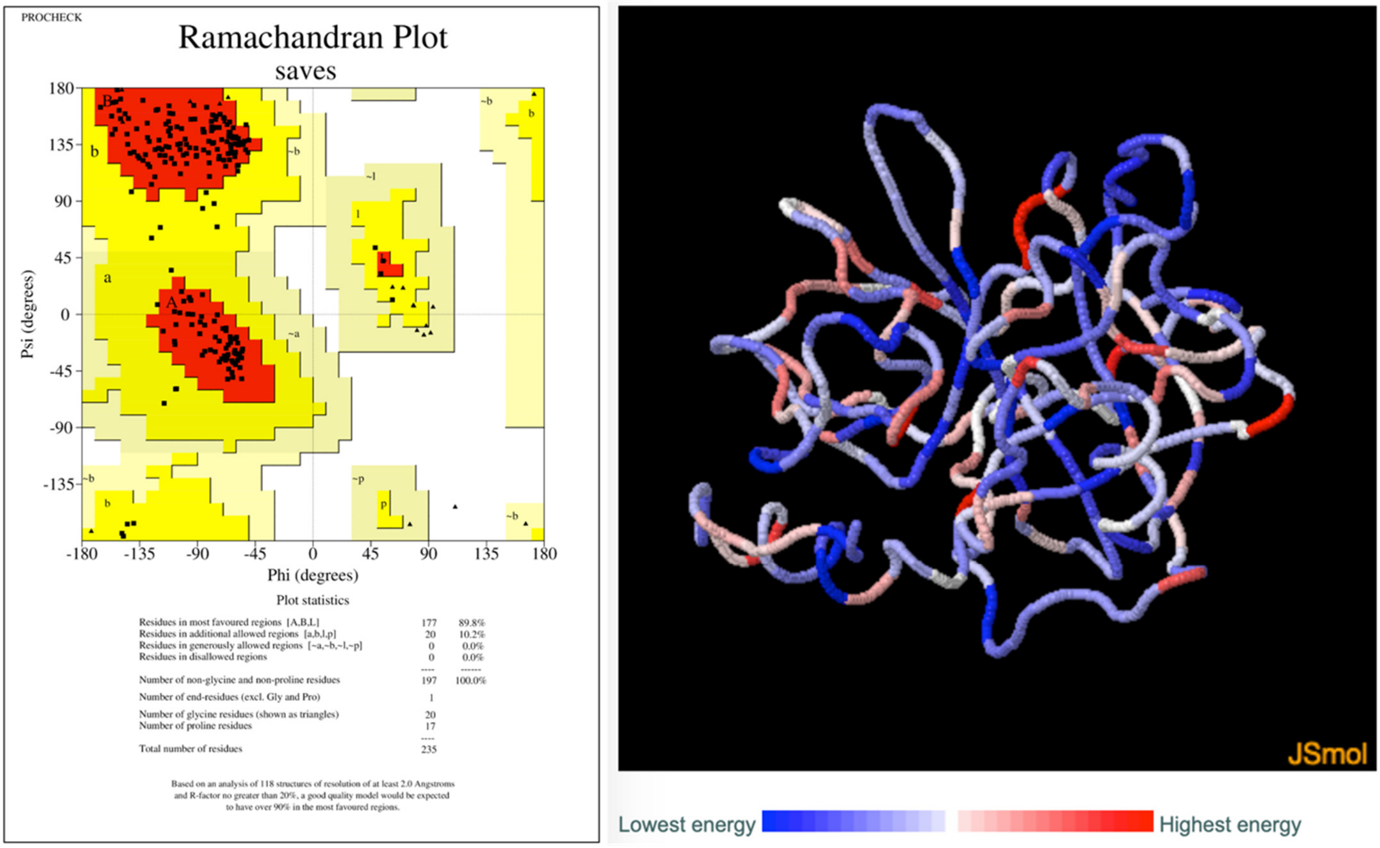

Figure S1. Stereochemical quality of the modeled serine proteinase using PROCHECK. **https://www.ebi.ac.uk/thornton-srv/software/PROCHECK/manual/parameters/manopt_01.html**

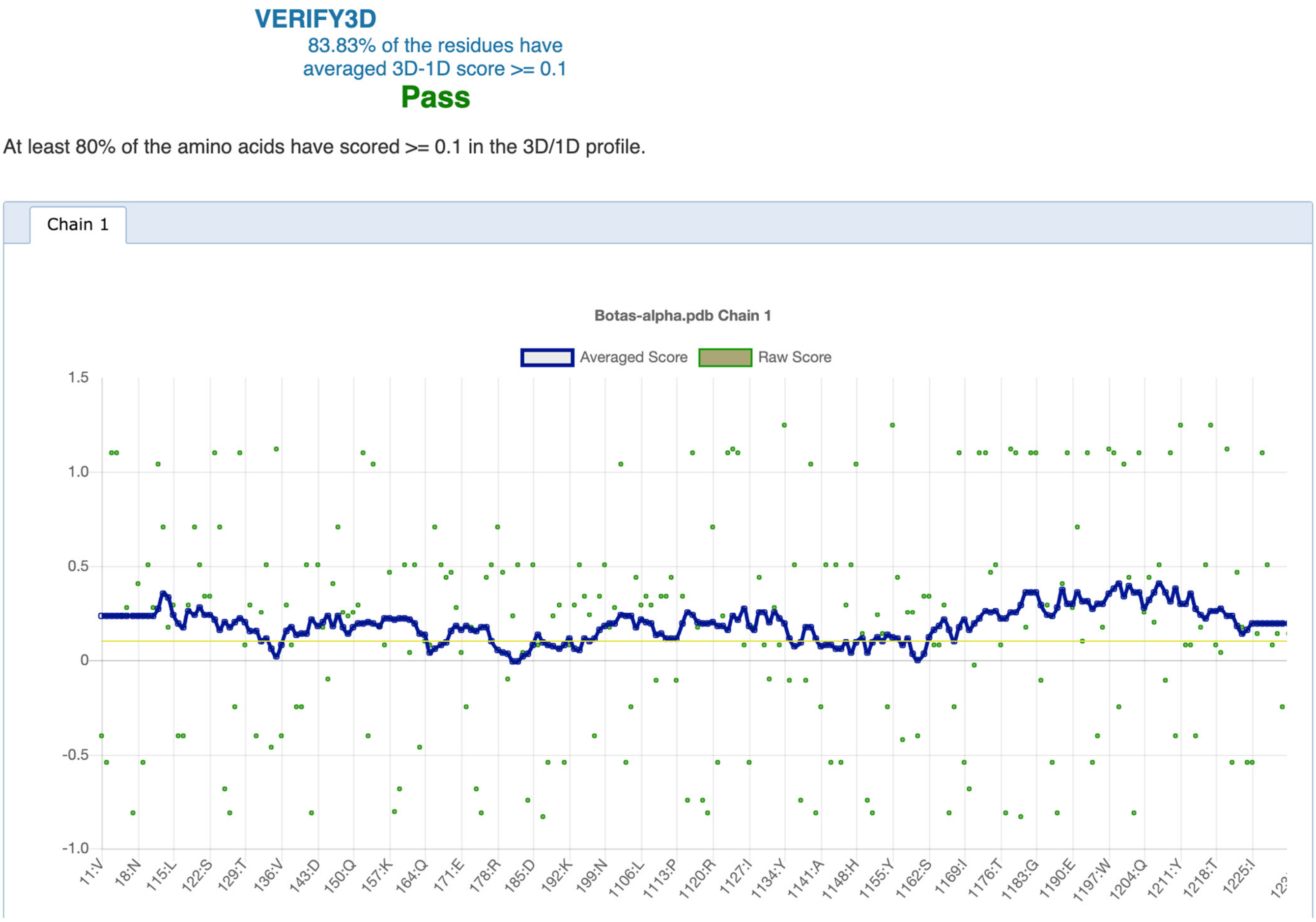

Figure S2. Compatibility of an atomic model (3D) with its amino acid sequence (1D).

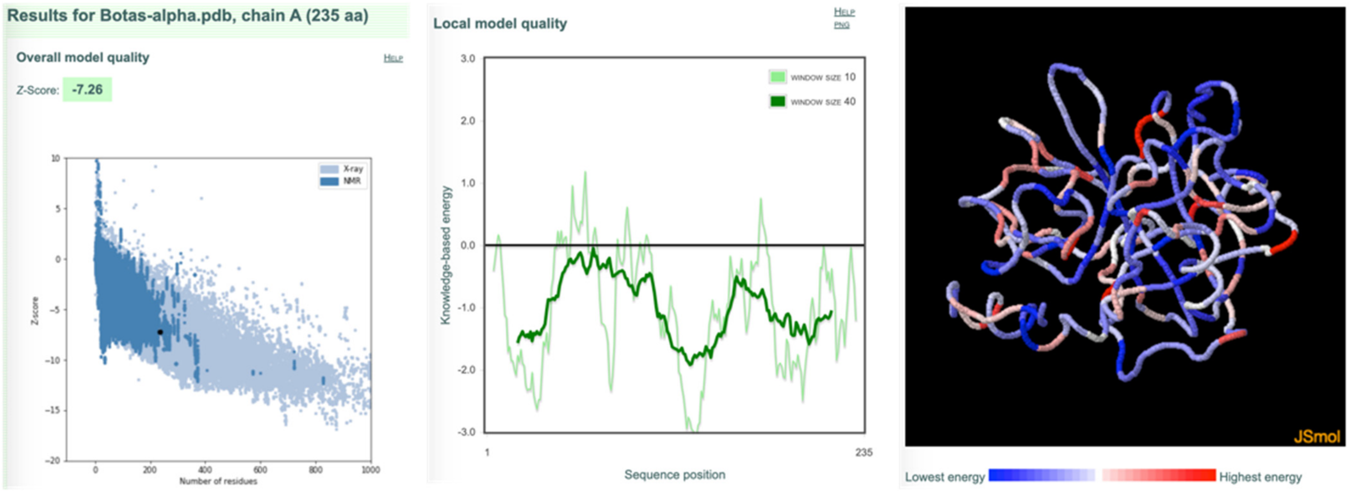

Figure S3. Energetic architecture of the modeled serine proteinase using the ProSA program.

Table S1. Mean effect size (Cohen's d), means, *p*-value, and 95% confidence intervals for the inhibitory activity of caffeine, caffeic, and chlorogenic acid on the pro-coagulant activity of *B. asper* venom

Caffeic acid	Chlorogenic acid	Caffeine	Positive control	
$\bar{x}$ (SEM)	$\bar{x}$ (SEM)	$\bar{x}$ (SEM)	$\bar{x}$ (SEM)	*p* value
64.7 (13.6)	241 (22)	31 (1.46)	23.6 (0.81)	0.001

		p value (Bonferroni)	Cohen's d-effect	Confidence Intervals (95%) Lower	Confidence Intervals (95%) Upper
Positive control	Caffeic acid	0.042	0.774	0.206	1.343
	Caffeine	1.00	0.139	−0.418	0.696
	Chlorogenic acid	<0.001	4.091	3.271	4.912

Table S2. Mean effect size (Cohen's d), means, *p*-value, and 95% confidence intervals for the inhibitory activity of caffeine, caffeic, and chlorogenic acid on the amidolytic activity of *B. asper* venom

Caffeic acid	Chlorogenic acid	Caffeine	Positive control	
$\bar{x}$ (SEM)	$\bar{x}$ (SEM)	$\bar{x}$ (SEM)	$\bar{x}$ (SEM)	*p* value
0.61 (0.03)	0.66 (0.02)	0.57 (0.02)	0.76 (0.001)	0.001

		p value (Bonferroni)	Cohen's d-effect	Confidence Intervals (95%) Lower	Confidence Intervals (95%) Upper
Positive control	**Caffeic acid**	0.001	−1.669	−2567	−0.772
	**Caffeine**	<0.001	−2.132	−3.073	−1.190
	**Chlorogenic acid**	0.063	−1.092	−1.948	−0.237

Table S3. Mean effect size (Cohen's d), means, *p*-value, and 95% confidence intervals for the inhibitory activity of caffeine, caffeic, and chlorogenic acid on the phospholipase A_2 activity of *B. asper* venom

Caffeic acid	Chlorogenic acid	Caffeine	Positive control	
$\bar{x}$ (SEM)	$\bar{x}$ (SEM)	$\bar{x}$ (SEM)	$\bar{x}$ (SEM)	*p* value
0.09 (0.004)	0.09 (0.004)	0.101 (0.005)	0.107 (0.004)	0.002

		p value (Bonferroni)	Cohen's d-effect	Confidence Intervals (95%) Lower	Confidence Intervals (95%) Upper
Positive control	**Caffeic acid**	0.004	−1.035	−16.353	−0.4341
	**Caffeine**	1.000	−0.372	−0.9503	0.2072
	**Chlorogenic acid**	0.012	−0.927	−15.232	−0.3317

Table S4. Mean effect size (Cohen's d), means, *p*-value, and 95% confidence intervals for the inhibitory activity of caffeine, caffeic, and chlorogenic acid on the proteolytic activity of *B. asper* venom

Caffeic acid	Chlorogenic acid	Caffeine	Positive control	
$\bar{x}$ (SEM)	$\bar{x}$ (SEM)	$\bar{x}$ (SEM)	$\bar{x}$ (SEM)	*p* value
0.377 (0.005)	0.363 (0.003)	0.364 (0.006)	0.353 (0.001)	0.002

		p value (Bonferroni)	Cohen's d-effect	Confidence Intervals (95%) Lower	Upper
Positive control	Caffeic acid	<0.001	12.384	0.6273	1.850
	Caffeine	0.327	0.5644	−0.0186	1.147
	Chlorogenic acid	0.447	0.5223	−0.0597	1.104

Table S5. Mean effect size (Cohen's d), means, *p*-value, and 95% confidence intervals for the inhibitory activity of green and roasted coffee extracts on the pro-coagulant activity of *B. asper* venom

Green Coffee	Roasted Coffee	Positive control	
$\bar{x}$ (SEM)	$\bar{x}$ (SEM)	$\bar{x}$ (SEM)	*p* value
**36.5 (1.98)**	**33.6 (2.98)**	**31.3 (0.29)**	**<0.001**

		p value (Bonferroni)	Cohen's d-effect	Confidence Intervals (95%) Lower	Upper
Positive control	Green Coffee	< 0.001	−5.4898	−7.33	−3.649
	Roasted Coffee	<0.001	−5.1761	−6.975	−3.377

Table S6. Mean effect size (Cohen's d), means, *p*-value, and 95% confidence intervals for the inhibitory activity of green and roasted coffee extracts on the amidolytic activity of *B. asper* venom

Green Coffee	Roasted Coffee	Positive control	
$\bar{x}$ (SEM)	$\bar{x}$ (SEM)	$\bar{x}$ (SEM)	*p* value
0.465 (0.04)	0.481 (0.04)	0.756 (0.00)	<0.001

		p value (Bonferroni)	Cohen´s d-effect	Confidence Intervals (95%) Lower	Upper
Positive control	Green Coffee	<0.001	−6.419	−8.11	−4.726
	Roasted Coffee	<0.001	6.064	4.45	7.681

Table S7. Mean effect size (Cohen's d), means, *p*-value, and 95% confidence intervals for the inhibitory activity of green and roasted coffee extracts on the phospholipase A_2 activity of *B. asper* venom

**Green Coffee**	**Roasted Coffee**	**Positive control**	
**$\bar{x}$ (SEM)**	**$\bar{x}$ (SEM)**	**$\bar{x}$ (SEM)**	***p* value**
**0.069 (0.008)**	**0.128 (0.019)**	**0.117 (0.004)**	**0.002**

		***p* value (Bonferroni)**	**Cohen's d-effect**	**Confidence Intervals (95%) Lower**	**Upper**
**Positive control**	**Green Coffee**	0.016	−1.102	−1.870	−0.333
	**Roasted Coffee**	1.000	−0.234	−0.998	0.530

Table S8. Mean effect size (Cohen's d), means, *p*-value, and 95% confidence intervals for the inhibitory activity of green and roasted coffee extracts on the proteolytic activity of *B. asper* venom

**Green Coffee**	**Roasted Coffee**	**Positive control**	
**$\bar{x}$ (SEM)**	**$\bar{x}$ (SEM)**	**$\bar{x}$ (SEM)**	***p* value**
**0.394 (0.008)**	**0.345 (0.018)**	**0.510 (0.003)**	**<0.001**

		***p* value (Bonferroni)**	**Cohen's d-effect**	**Confidence Intervals (95%) Lower**	**Upper**
**Positive control**	**Green Coffee**	<0.001	−2.38	−3.251	−1.50
	**Roasted Coffee**	<0.001	3.39	2.381	4.41
